# Auto-Global Examination of Mental State (Auto-GEMS): a web-based self-administered cognitive screening

**DOI:** 10.1007/s40520-024-02862-z

**Published:** 2024-11-11

**Authors:** Veronica Pucci, Giulio Contemori, Maria Silvia Saccani, Giorgio Arcara, Sara Mondini, Mario Bonato

**Affiliations:** 1 Department of Philosophy, Sociology, Education and Applied Psychology, Department of Developmental and Social Psychology and Socialization, and Human Inspired Technology Centre (HIT), University of Padua, Padua, Italy; 2 Department of General Psychology, University of Padua, Padua, Italy; 3 Padua Neuroscience Centre, University of Padua, Padua, Italy; 4 IRCSS, San-Camillo Hospital, Venice, Italy

**Keywords:** Tele-neuropsychology, Computerized screening, Self-administration, Digital health, Web-based cognitive screening

## Abstract

Recent methodological developments have contributed to a significant advance in computerised neuropsychological instruments and procedures, including those accessible from remote. In this paper we present Auto-GEMS, a newly developed, web-based, self-administered screening test allowing to quickly estimate an individual’s cognitive state also considering their cognitive reserve. Auto-GEMS measures cognitive functioning on eleven items similarly to the in-person paper-and-pencil version (GEMS) and to the remote (phone or video call) version (Tele-GEMS) of the same screening. We collected normative data on a sample of 1308 Italian-speaking participants (age range 18–93) to verify its psychometric properties and computed regression models on demographic variables to establish clinical cut-offs. The psychometric properties of Auto-GEMS have shown good internal consistency, test-retest reliability and convergent validity. This short and user-friendly tool has a number of potential applications. For instance, it can be useful in clinical practice to monitor the cognitive profile of patients or vulnerable individuals, or even administered in a face-to-face, standard clinical setting. It can also be used in research studies to screen participants. The testing materials and the collected data are freely available in a digital archive along with a web App to visualise the test outcome with reference to its normative data.

**Supplementary Information:**

The online version contains supplementary material available at 10.1007/s40520-024-02862-z.

## Introduction

Over the years, tools for cognitive and neuropsychological assessment have been continuously developed and improved in parallel with technological innovation. A major drive in this process was the Covid-19 pandemic, which boosted the implementation of tele-neuropsychology as complementary to traditional in-person assessments [3]. Tele-neuropsychology is defined as the application of audiovisual technologies that allow researchers and clinicians to conduct remote neuropsychological assessments [19]. It has a number of advantages as well as several disadvantages with respect to standard, in-person neuropsychological assessment [16, 18, 31]. The main advantage is that tele-neuropsychology can, broadly speaking, allow more persons to undergo a cognitive assessment and, ideally, to get in touch with a neuropsychologist. It can, for instance, facilitate the assessment of individuals who need longitudinal follow-up or who cannot move from their homes or are living in particularly isolated areas [4] or are not independent when they need to reach a hospital. Additional advantages have been described in terms of ease of administration, more objective scoring, more consistent user experience [5], possibility of parallel testing, and better match with normative data (Parson et al., 2018). At the same time, tele-neuropsychology is characterised by several limitations, the overarching one consisting in reduced control by the neuropsychologist [27, 34] with respect to classical settings. Additional, commonly reported, disadvantages are related to hardware and software issues like lack of precision, variability and obsolescence [16, 18, 31]. The picture on the pros and cons of neuropsychology is heterogeneous also when considering emotional aspects: while being tested in a familiar place and with their own device no doubt results in less anxiety and better performance, the need to deal with technological devices might induce anxiety for those persons less familiar with the use of a computer [27, 31].

Research indicates that tele-neuropsychological evaluation is reliable, also when testing older adults who might be less familiar with Information and Communication Technologies [21]. However, information gathered remotely cannot be considered equivalent to that obtained face-to-face [26]. Indeed, in a remote setting, no qualitative evaluation and no information about how the examinee has carried out the test can be available.

Another core characteristic of tele-neuropsychology is that its instruments are almost invariably heterogeneous. This heterogeneity is not only due to the diversity of cognitive functions to be measured, but also related to the fact that some tools are more suitable than others for remote administration [22]. It seems worth noting that many tele-neuropsychological tools have been specifically normed for use via telephone or videoconference. According to the review by Zanin and colleagues [38], almost invariably the few tele-neuropsychological tools available in Italian in 2022 required a clinician for being administered as they were based either on the telephone (e.g. Itel-MMSE; Metitieri et al., [25]; for a more recent tool see t-FAB; Aiello et al., [1, 2, 15]) or on web-streaming. The relative lack of web-based self-administered tools described for Italy by Zanin et al., [38], also characterises other countries (see the review by Chan et al., [6]). The advantages of self-administered, web-based tests include, for instance, a great potential for longitudinal group studies in healthy older adults to monitor their potential decline (for a review, see Tsoy et al., [36]). While hundreds of computerized self-administered tools have been developed, only very few are available for clinical use. In a literature search focused on cognitive self-testing, after a first selection of 3000 papers Charalambous et al. [7] included in their review fewer than 40 tests/tools for cognitive decline available for free or at low cost on the web or on Apps. For the majority of these tests, it was not possible to access the normative data or psychometric properties because they were not included in the published studies [7]. Yet, it would be a mistake to assume that all computerised, self-administered tests have poor psychometric properties. Some of these tests are inspired by traditional ones (e.g., Cogstate by Maruff et al., [23] and are characterised by high levels of reliability and validity, with results comparable to those obtained with their paper-and-pencil counterparts [11]. Some of them are used in a wide range of contexts, both clinical and non-clinical, showing their potential and efficacy.

The review of Tsoy and colleagues [36] reported ten self-administered screenings available worldwide and validated to detect MCI in older adults. Among these tools, in the USA, Vyshedskiy and colleagues [37] developed a brief, self-administered web-based cognitive test for remote longitudinal monitoring: the Boston Cognitive Assessment (BOCA). In Brazil, Memoria and colleagues [24] adapted the Computer-Administered Neuropsychological Screen for Mild Cognitive Impairment (MCI) and dementia of Alzheimer’s type. In China, Wong and collaborators developed the Computerised Cognitive Screen (CoCoSc) Hong Kong version. In Australia, Maruff et al. [23] adapted and validated the CogState Brief Battery (CBB) to assess cognitive changes in the preclinical stages of dementia in persons with MCI and with Alzheimer’s disease.

However, to the best of our knowledge, none of these has been translated into Italian and normed for the Italian population, and no other self-administered screening seems to be available in Italian. Only very recently Panzavolta and colleagues [30] presented a digital platform in Italian for the diagnosis of MCI. They validated the tool by using avatars representing clinical cases and therefore no normative data were released.

We therefore intend to provide a new, web-based, self-administered tool for speedy cognitive screening: Auto-GEMS (Auto-Global Examination of Mental State). Auto-GEMS is open and free-to-use and is provided here with its access links, its psychometric properties and norms for the Italian population. Auto-GEMS is the computer-based, self-administered version of the in-person screening GEMS [27] which is complemented by the remote screening Tele-GEMS [28]. Auto-GEMS can be self-administered with or without supervision either online or in person, depending on the clinical need and on the research question being addressed. This allows to temporally dissociate test administration from the interaction with a clinician, therefore leading to great flexibility in terms of administration commitment on both sides.

It seems worth reiterating that to overcome the limitations characterising self- administration, computer-based tools may also be useful during a traditional in-person assessment under the direct observation of a neuropsychologist to evaluate examinees’ independence and their ability to interact with the computer.

In line with this, the “hybrid” model emphasises the need to maximise the benefits of tele-neuropsychology and traditional methods by integrating and aggregating information collected with digital and paper-and-pencil tools [35]. The hybrid model in neuropsychology integrates traditional assessments with novel remote methodologies, incorporates digital tools into clinical practice, and encourages collaboration with experts from various disciplines. This approach aims to update the field with technology-based practices to enhance the quality of evaluations, improve patient care, and ensure that neuropsychology keeps up with the most recent technological developments [35].

## Materials and methods

### Participants

A sample of 1308 healthy Italian individuals (779 females, 60% of the total) was recruited for this study. All participants were over 18 y.o., were native Italian speakers, and had no diagnosis of psychiatric or neurological diseases. They took part voluntarily in the study and were all contacted directly or answered advertisements. The authors were able to control the demographic information and match the inclusion criteria. Table 1 shows their stratification by age and education. Participants were born/living in different geographical areas of Italy. The mean age of the sample was 51.2 years (SD = 18), ranging from 18 to 93 y.o., and their mean education was 13.3 years (SD = 4.1), ranging from 5 to 21 years.

All of them gave their informed consent to participate in the study and allowed us to access their scores. They were aware that they could stop and withdraw from the test at any time while performing it. This study was approved by the Ethical Committee of the School of Psychology, University of Padua, and adheres to the Declaration of Helsinki.

**Table 1:** The table shows the stratification of the normative sample tested online with Auto-GEMS according to age and education (in years)

Age	Education						**Total**
	0–5	6–8	9–13	14–16	17–18	> 18	
18–30	1	4	115	110	42	14	**286**
30–40	0	0	16	18	21	23	**78**
40–50	1	14	38	15	20	13	**101**
50–60	4	107	215	68	68	18	**480**
60–70	8	44	67	15	42	10	**186**
70–80	37	34	24	9	14	0	**118**
> 80	37	13	5	1	2	1	**59**
**Total**	**88**	**216**	**480**	**236**	**209**	**79**	**1308**

## Method

Auto-GEMS is a web-based cognitive screening tool developed using JavaScript, CSS, and HTML, designed to be compatible with current web browsers. The framework utilised for its development is jsPsych version 6.3, specifically tailored for creating behavioural experiments within web-browsers [12, 13]. This platform employs plugins to define various events such as image display and to collect responses including key presses and their timestamps, organising them into a timeline for streamlined data collection. It allows a flexible use in different functioning modalities, both online and offline.

During the validation phase, data collection was conducted remotely via the web-based interface. A link was generated and sent to the participants via e-mail. They autonomously completed the screening, and upon assessment completion, their data were automatically sent back and stored on the server of the Department of General Psychology at the University of Padua. This approach allowed automatic data collection without requiring any additional action from the participant. This is the functioning mode (Remote Data Collection) to choose when data need to be collected from remote, for example, in case of a research project. To implement this modality, researchers must download the code from a digital repository (link at the end of the paragraph).

Other two main modalities are available for using Auto-GEMS: the Local Functioning Modality and the Ready-to-Use Version.

In the Local Functioning Modality, data are saved only on the computer running the test (e.g. the clinician’s or experimenter’s computer) without requiring an internet connection. Finally, the Ready-to-Use Version is accessible via a specific link (https://unipd.link/auto-GEMS). Upon clicking on it, the testee’s web browser connects to the server hosted by the Department of General Psychology, University of Padua, to download the necessary code and files. During this initial connection, an anonymous log data is generated on the server, contributing to monitoring and analytics. Once downloaded, the screening operates only locally, on the participant’s device. Importantly, similarly to the Local Functioning Modality also in the Ready-to-Use Version no sensitive data is uploaded or stored on the server upon completion of the screening. Data files in .csv format are saved solely on the participant’s device, ensuring privacy and compliance with data protection regulations.

For both the Local Functioning Modality and the Ready-to-Use Version, Auto-GEMS allows the comparison of individual screening scores with normative data through a dedicated Shiny app (https://gcontemori.shinyapps.io/auto-gems_shiny/*).* In the Local Functioning Modality, clinicians can access the .csv file generated upon completion of the screening to extract Auto-GEMS scores, Cognitive Reserve (CR) scores, and participants’ ages. This information can be then uploaded into the Shiny app to visualise and compare the results against the normative data we collected and made available (see later), thus aiding in clinical interpretation and decision-making. In the Ready-to-Use Version, after the test is completed, the user is automatically redirected to the Shiny app, where the normative data comparison is displayed without requiring any user input.

As a workspace we used JATOS, an open-source platform that operates under the Apache 2 Licence, and its source code can be found on GitHub, as detailed by Lange et al. [20]. JATOS facilitates the rapid configuration of servers and the deployment of tests. The JATOS manual can be found at https://www.jatos.org/Get-started.html.

The JATOS workspace can be created on a server or in a cloud-based environment. It can also be set up on the clinician’s or experimenter’s computer (local server). As already mentioned, once the .jzip is downloaded and imported in the local JATOS instance, the test can run in the browser even without a web connection. This approach allows the clinician/experimenter to directly administer the test to the participant, even if it restricts the remote use of the test. Conversely, when imported on a global server, Auto-GEMS enables researchers and clinicians to create and share the test link with third parties and collect individual data remotely.

The source code of Auto-GEMS is “ready to use” and importing the .jzip file into a pre-configured JATOS server needs only a one-click operation. This approach offers the advantage of securely storing participant data on researchers’ servers (either local or global), ensuring complete data control and safeguarding participant confidentiality and ethical considerations.

Auto-GEMS is freely accessible under a Creative Commons licence, and its source code is available on the Open Science Framework (OSF) repository: https://osf.io/fq8g7/ in compliance with OPEN science good practices. Refer to the link above also for potential, future, project developments (e.g. Auto-GEMS standardization in other languages).

## Materials

In the current study, we have administered four distinct instruments (all in Italian, different formats):

1. Auto-Global Examination of Mental State (web-based);
2. The Global Examination of Mental State (GEMS, Mondini et al., [27] paper-and-pencil);
3. The Tele-Global Examination of Mental State (Tele-GEMS, Montemurro et al., [28] telephone-based);
4. The Cognitive Reserve Index questionnaire (CRIq, Nucci et al., [29] in presence).

1) Auto-GEMS (Auto-GEMS-A and Auto-GEMS-B) Auto-GEMS (in two parallel versions A and B) first collects examinees’ demographic information and quantifies their cognitive reserve through a shortened version (six items) of the Cognitive Reserve Index questionnaire adapted from Nucci et al. [29]. Information about the level of education (CR-Education), occupation (type and years of working; CR-WorkingActivity) and type and frequency of free time activities (CR-LeisureTime) is collected. CR-Total is the average of the three subscores. Subsequently, a brief audio check is presented, followed by 11 tasks in a fixed order, each measuring a different aspect of cognition. Auto-GEMS (A and B) requires about 10 min to be completed and includes the following tasks:

*Orientation*. To evaluate the capacity of orientation in time and space, three questions are asked: Which season are we in? What year is it? Relative to Venice, is Rome located to the North, South, East or West? The system automatically records the date of administration and, thus, can recognise the correct answers. One point is assigned to each correct answer.

*Immediate memory*. To evaluate verbal short-term memory, six words are at the same time presented both in written and auditory form. After their presentation, the examinee has to type the words they remember in the given gaps, regardless of the order of presentation. One point is assigned to each correct word reported.

*Months backwards*. To evaluate working memory, the examinee is asked to write the months of the year backwards, starting from October and skipping one month at a time (i.e., October, August, June, and so on five times). One point is assigned to each correct answer.

*Puzzle*. To evaluate visuo-constructional abilities, the examinee is asked to use the mouse via drag and drop to rebuild a figure (i.e., a train) cut into four pieces. One point is assigned to each piece in the correct position.

*Spatial representation*. To evaluate spatial abilities, the examinee is asked to decide whether the two hands of an imaginary clock are placed on the two opposite sides of the clock, or both on the right-hand side, or both on the left-hand side. One point is assigned to each correct answer.

*Delayed memory*. To evaluate the verbal long-term memory, the examinee is asked to recall the six words previously presented (around five minutes later) and write those they remember in the given gaps. One point is assigned to each correct word reported.

*Naming*. To assess language and lexical access, the examinee has to write the name of four non-living objects (i.e., pear, table, compass and saxophone) presented to them one picture at a time. One point is assigned to each correct answer.

*Comprehension*. To verify the comprehension of a simple order, the examinee is asked to perform a three-step command by using the keyboard: “*Press the letter ‘A’ twice after pressing the letter ‘B’ once*”. The command is presented both verbally and in written form. One point is assigned to each correct execution.

*TMT-A*^1^. To assess processing speed and attention, the examinee is asked to click on a set of numbers (from 1 to 14) in ascending order as quickly as possible. The task is structured similarly to the original one [33]. Both accuracy and processing speed (in seconds) are recorded automatically and counted for scoring.

*TMT-B*^1^. To assess divided attention, cognitive flexibility and processing speed, the examinee is asked to click on a set of numbers (from 1 to 7) and letters (from “A” to “G)” in ascending and alphabetical alternating order as quickly as possible. Both accuracy and processing speed (in seconds) are recorded and taken into account for scoring.

*Metaphor*. To evaluate pragmatic abilities with figurative language, the examinee is asked to read a sentence with a metaphoric meaning (“*Today I visited the town library. That archive is a mine!*”) and then choose among three possible explanations of the sentence. One point is assigned if the answer is correct.

The two parallel versions have the same structure of tasks, and they only differ in item content.

Each of the 11 tasks of Auto-GEMS (A and B) results in a raw score, which is then proportionally recorded in such a way that each task (representing mainly one cognitive function) weighs the same on the global score. For example, if a participant obtained 3/6 as a raw score in the Immediate memory task, the proportion will be 3*9/6 = 4.5. Each weighted score ranges from 0 to 9, and the total score is calculated by summing the scores of each single task.

2) GEMS is a paper-and-pencil cognitive screening [27] made up of eleven subtasks: Orientation; Immediate memory; Months backwards; Puzzle; Clock; Delayed memory; Picture naming; Verbal comprehension; Visual attention; Fluency; Metaphor comprehension. GEMS was administered in person (duration about 10 min).

3) Tele-GEMS is a cognitive screening test administered remotely, by telephone or videoconference [28]. Tele-GEMS includes ten tasks: Orientation, Immediate memory recall, Months backwards, Spatial representation, Naming, Delayed memory, Verbal comprehension, Auditory attention, Fluency, and Metaphor comprehension. Its administration by telephone lasted about 10 min.

4) Cognitive Reserve Index questionnaire ([29], CRIq, available at https://www.cognitivereserveindex.org/index.html) measures a person’s CR considering education, working activity, and leisure time activities during the lifespan. CRIq was administered in presence by an examiner, and it lasted about 10 min.

### Procedure

**Fig. 1:**
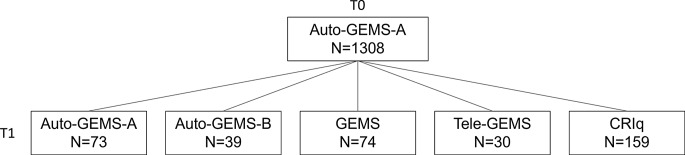
The Figure describes the data collection design of the present study. The upper box represents data collected at T0 by using Auto-GEMS, while the lower ones represent data collected at T1 (4–6 weeks after T0)

Each person recruited was preliminarily interviewed to verify inclusion criteria and was informed about the aim and procedure of the research. The link to Auto-GEMS-A was then sent to each participant via email. After one month, a sub-sample (*N* = 73) was re-tested with the same version of Auto-GEMS (i.e., Auto-GEMS-A), while another sub-sample (*N* = 39) was re-tested with the parallel version B of Auto-GEMS (from now on Auto-GEMS-B). Other two sub-samples were also assessed either with GEMS (*N* = 74) or Tele-GEMS (*N* = 30) after a time interval of 4–6 weeks. A sample of 159 participants was also assessed with the complete version of the Cognitive Reserve Index questionnaire [29] to verify the correlation with the six items about cognitive reserve selected and proposed in Auto-GEMS. Figure 1 shows the data collection design.

### Statistical analyses

The analyses were performed with R software (version 4.1.0; R Core Team [32]. Convergent validity of Auto-GEMS was assessed by Pearson’s correlations with GEMS and Tele-GEMS. Internal consistency was calculated through a standardised Cronbach’s alpha on all items. Test-retest and parallel-form reliability were analysed through Pearson’s correlations. The practice effect was calculated using paired sample t-tests. We also provided significant change thresholds with a regression-based approach [10]. Exploratory factor analysis was performed to investigate the underlying factor structure of the data. Given the different subtests included in Auto-GEMS with respect to GEMS and Tele-GEMS it was not possible to run a confirmatory factor analysis. We therefore decided to run a factor analysis to better elucidate the pattern of correlations between tasks in Auto-GEMS.

We assessed the relationship of age, education, and CR with Auto-GEMS by multiple regressions, with the total Auto-GEMS score as the dependent variable. Cut-offs were obtained following Crawford and Garthwaite’s (2006), a regression-based approach that overcomes the limits of arbitrary thresholds of age, and education values [27], and that takes into account the difference between sample and population.

## Results

The mean cognitive reserve score measured in Auto-GEMS-A was 123.3 (SD = 16.6; ranging from 74 to 208). Preliminary analysis showed that CR section of Auto-GEMS-A highly correlated with the original CRIq by Nucci et al., [29] (CR-Total: *r*(157) = 0.80; CR-Education: *r*(157) = 0.82; CR-WorkingActivity: *r*(157) = 0.83; CR-LeisureTime: *r*(157) = 0.59). Auto-GEMS-A mean score across all participants was 81.8/100 (SD = 11.4; range 27.8–100). The distribution was left-skewed, with no ceiling or floor effects (see Table 2 for detailed descriptive statistics).

**Table 2:** Descriptive statistics of the normative sample. Descriptive statistics are reported for demographic variables (age and education, in years), Cognitive Reserve, and for each Auto-GEMS task as well as for its global score

	Mean	SD	Median	Min	Max	Kurtosis	Skewness	Q1	Q3
Age	51.2	18	55	18	93	-0.8	-0.21	34	62
Education	13.3	4.1	13	5	21	-0.6	-0.4	13	16
CR	123.3	16.6	122	74	208	1.4	0.5	122	134
Orientation	7.8	1.7	9	0	9	1	-1.23	6	9
Immediate memory	7.2	1.8	7.5	0	9	0.6	-0.9	6	9
Months backwards	8.1	1.8	9	0	9	6.7	-2.5	7.2	9
Puzzle	8.9	1.8	9	3	9	101.5	-9.7	9	9
Spatial Representation	8.6	0.5	9	0	9	16.6	-3.4	9	9
Delayed Memory	6.1	2.3	6	0	9	-0.4	-0.5	4.5	7.5
Naming	8.4	1.1	9	4.5	9	1.4	-1.5	9	9
Comprehension	7.6	2.6	9	0	9	0.9	-1.5	6	9
TMT-A	4.9	2.8	5	0	9	-1.2	-0.1	2	7
TMT-B	4.7	2.9	5	0	9	-1.2	0	2	7
Metaphor	8.5	2.1	9	0	9	12.9	-3.9	9	9
Auto-GEMS total	81.8	11.4	83.5	27.8	100	1.6	-1.1	75.5	90.2

### Internal consistency

The internal consistency, calculated using a standardised Cronbach’s alpha on all items, was high (alpha = 0.72). Each item showed a significant correlation with the global score: *r* = 0.30 for Orientation, *r* = 0.69 for Immediate memory, *r* = 0.44 for Months backwards, *r* = 0.24 for Puzzle, *r* = 0.41 for Spatial representation, *r* = 0.68 for Delayed memory, *r* = 0.34 for Naming, *r* = 0.46 for Comprehension, *r* = 0.75 for TMT-A, *r* = 0.73 for TMT-B and *r* = 0.45 for Metaphor.

### Convergent validity

**Fig. 2:**
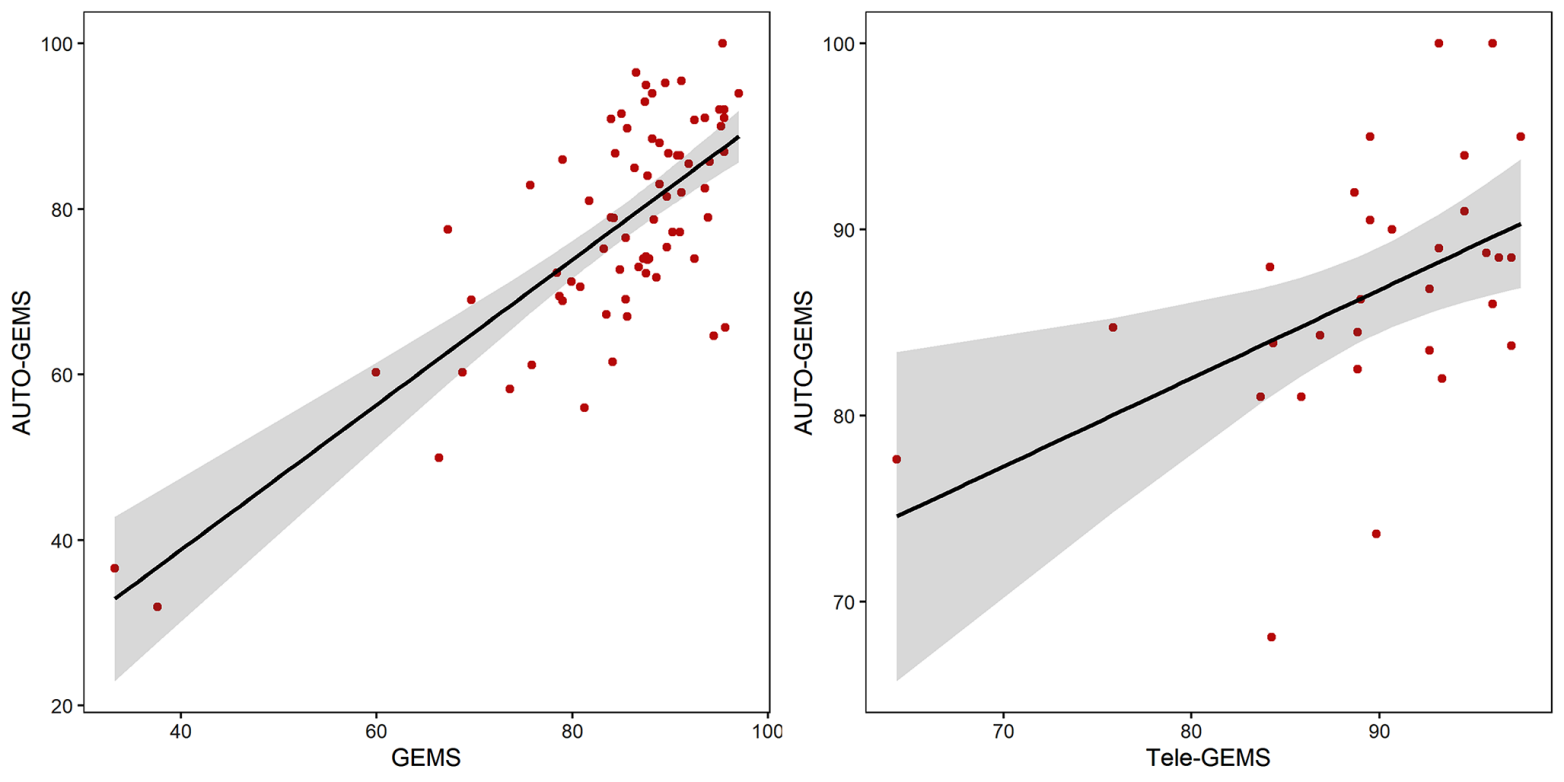
The figure shows the correlation between Auto-GEMS and GEMS (left panel) or Auto-GEMS and Tele-GEMS (right panel)

In order to verify the capacity of Auto-GEMS-A to capture the global cognitive functioning, two sub-samples of participants (*N* = 74) were also assessed with GEMS [27] and (*N* = 30) with Tele-GEMS [28], two available screening tests that have been already shown to reliably measure overall cognitive function. Auto-GEMS-A showed a relatively large correlation with the more similar GEMS (*r* = 0.74) and a moderate correlation with the relatively different Tele-GEMS (*r* = 0.49), (see Fig. 2).

### Test-retest reliability, practice effect and equivalence of parallel forms

The test-retest reliability of Auto-GEMS-A was calculated through Pearson’s *r* in a sub-sample of 73 participants assessed twice within a four-week interval. The correlation coefficient for the Auto-GEMS-A global score was very high (*r* = 0.88, *p* < 0.001), and for the single tasks the coefficient ranged from 0.44 to 0.85 (see Table 3 for more details).

The practice effect of Auto-GEMS-A after repeated administrations was evaluated with paired sample t-tests by comparing the two total scores of the same version. The significant effect obtained [t(72) = 3.26, *p* = 0.002] indicates that the second time participants performed significantly better than the first (mean = 78.48 vs. mean = 80.67) with a mean difference of 2.19. The practice effect on each single task was not significant, except for the Immediate and Delayed memory tasks (see Table 3).

A sub-sample of 39 participants was assessed with both versions of Auto-GEMS (A and B) with a four-week interval, and the two parallel forms showed a good correlation (*r* = 0.55, *p* < 0.001). The analysis using Auto-GEMS-B at retest did not show evidence of practice effect (See Table 3).

**Table 3:** The table reports the test-retest reliability (Pearson’s *r*) and practice effect (paired sample t-tests) of single task scores and the global score of both re-tests with Auto-GEMS-A and Auto-GEMS-B

Tasks and total score	Test retest-reliability Pearson’s *r*		Mean difference		Practice Effect Paired sample t-test	
	Auto-GEMS-A vs. Auto-GEMS-A	Auto-GEMS-A vs. Auto-GEMS-B	Auto-GEMS-A vs. Auto-GEMS-A	Auto-GEMS-A vs. Auto-GEMS-B	Auto-GEMS-A vs. Auto-GEMS-A	Auto-GEMS-A vs. Auto-GEMS-B
Orientation	0.44	0.71	0.25	-0.15	t(72) = 1.51 *p* = 0.25	t(38)=-0.57 *p* = 0.57
Immediate memory	0.62	0.40	-0.84	-0.54	t(72)=-4.67 *p* < 0.01	t(38)=-1.4 *p* = 0.169
Months backwards	0.49	0.30	-0.37	0.14	t(72)=-1.42 *p* = 0.159	t(38) = 0.238 *p* = 0.813
Puzzle	1	1	-0.08	0	t(72)=-1.42 *p* = 0.159	-
Spatial representation	0.50	0.72	0.03	0.29	t(72) = 1.16 *p* = 0.87	t(38) = 1.3 *p* = 0.201
Delayed memory	0.66	0.27	-1.01	-0.81	t(72)=-4.4 *p* < 0.001	t(38)=-1.53 *p* = 0.134
Naming	0.56	0.40	0.12	-0.17	t(72) = 1.16 *p* = 0.251	t(38)=-1.14 *p* = 0.262
Comprehension	0.48	0.30	-0.37	-0.15	t(72)=-1.14 *p* = 0.26	t(38)=-0.25 *p* = 0.803
TMT-A	0.80	0.33	0.03	0.42	t(72) = 0.13 *p* = 0.895	t(38) = 0.78 *p* = 0.437
TMT-B	0.76	0.10	-0.07	0.29	t(72) = 0.34 *p* = 0.732	t(38) = 0.69 *p* = 0.495
Metaphor	0.85	0.34	0.12	0.92	t(72) = 1 *p* = 0.231	t(38) = 1.43 *p* = 0.16
Total score	0.88	0.54	-2.19	0.24	t(72) = 3.26 *p* = 0.002	t(38) = 0.09 *p* = 0.926

### Factor analysis

The purpose of the factor analysis was to examine the relationships among the subtests of Auto-GEMS and to explore significant patterns of association. The goodness of fit was satisfactory with four factors ($\:\chi\:2$=20.42, df = 17, *p* = 0.254), indicating that they are sufficient to explain the data structure. The factor loadings suggest that the first factor (Factor 1) is probably linked to processing speed and attention, being that TMT-A and TMT-B have the highest loadings. The second factor (Factor 2) may be associated with memory, as both immediate and delayed memory tasks have the highest loadings. The third factor (Factor 3) is probably associated with verbal working memory capacity as the months backwards task has the highest loadings. The fourth factor (Factor 4) is (weakly) represented by all tasks of Auto-GEMS, and possibly represents global cognition. See Table 4 for more details.

**Table 4:** Factor analysis for all Auto-GEMS tasks. The loadings for each of the subtests of Auto-GEMS indicate the contribution of each task to the factor. Dashes refer to loadings smaller than 0.1. Values in bold indicate those loadings which are 0.5 larger than the other values within the same factor

Tasks	Factor 1	Factor 2	Factor 3	Factor 4
Orientation	-	-	-	0.242
Immediate memory	0.205	**0.851**	-	0.266
Months backwards	0.101	0.118	**0.972**	0.163
Puzzle	-	-	-	0.366
Spatial representation	-	-	0.140	0.466
Delayed memory	0.221	**0.753**	-	0.218
Naming	-	-	-	0.328
Comprehension	0.151	-	-	0.266
TMT-A	**0.965**	0.180	-	0.171
TMT-B	**0.724**	0.233	-	0.183
Metaphor	0.130	0.169	-	0.291

### Thresholds for significant change

Using the regression-based approach [10], we calculated thresholds to identify significant change after repeated administrations of both Auto-GEMS versions (A and B). This method allows determining whether the score obtained on the second measurement is significantly different from the predicted one, thus indicating a significant change (improvement or worsening). Thresholds for significant change are available for the administration of Auto-GEMS-A followed by either the same version (Auto-GEMS-A) or the parallel one (Auto-GEMS-B). Supplementary Table 1 can be used to determine whether a significant change has occurred in the same individual after a second administration of Auto-GEMS-A.

### Relationship with demographic variables

As expected, Auto-GEMS global score negatively correlates with Age (*r*=-0.59; *p* < 0.001), positively with Education (*r* = 0.53; *p* < 0.001) and Cognitive Reserve (*r* = 0.27; *p* < 0.001). No difference between males and females was observed (t = 0.926, *p* = 0.354).

A series of regression models was then computed to quantify the effect of Age, Education, CR and Sex on the Auto-GEMS-A score and to derive the appropriate cut-off scores (see paragraph below). We entered one predictor at a time and, based on the Akaike Information Criterion, we chose the one with the best fit. The model with all four predictors (Age, Education, CR and Sex - Model 4) had the best fit. See Table 5 for more details.

**Table 5:** Table 5 shows the linear regression models which tested the effect of the demographic variables (Age, Education, CR, Sex) on Auto-GEMS-A. The second column reports the predictors entered in the model; the third, the values of the model coefficient (Beta and *p*); the fourth, the model fit measures (R^2^, F-test and associated *p*-value and Akaike Information Criterion)

Dependent variable: Auto-GEMS total score						
Model	Predictor(s)	Model coefficients		Model fit measures		
		Beta	*p*	R^2^	F test (*p*)	AIC
Model 1	Age	-0.37	< 0.001	0.344	685.4 (< 0.001)	9541
Model 2	Age	-0.28	< 0.001	0.434	500.6 (< 0.001)	9350
	Education	0.95	< 0.001			
Model 3	Age	-0.29	< 0.001	0.439	339.4 (< 0.001)	9335
	Education	0.81	< 0.001			
	CR	0.05	0.001			
Model 4	Age	-0.29	< 0.001	0.441	256.9 (< 0.001)	9331
	Education	0.81	< 0.001			
	CR	0.05	0.002			
	Sex	-1.14	0.018			

### Cut-off scores

Cut-offs were calculated using the regression-based approach by Crawford and Garthwaite [10]. This method predicts an examinee’s score from demographic variables of interest (i.e., Age, Education, CR, and Sex). These variables were identified as relevant by comparing different regression models and choosing the one with the best fit. An essential feature of this method is that it is specifically designed to compare a single case to a matched sub-sample with the same characteristics. Importantly, these cut-offs can be interpreted as the probability of observing a performance equal to or lower than the one observed, taking into account the uncertainty that data derives from a sample drawn from a larger population.

### Feedback from participants

After performing Auto-GEMS (A and B), participants could provide feedback on their experience with the test by selecting one or more statements (Items are reported in Supplementary Table 2). A total of 881 out of 1308 participants gave their feedback: 70% of respondents stated that they performed the test to the best of their ability; 63% declared that they carried out the tasks autonomously from start to end; 16% of respondents stated that they carried out the task autonomously, but a person helped them with mouse and keyboard; 15% were interrupted or distracted during the test; 14% stated that a person opened the email for them and then they carried out the task autonomously; 8% stated they carried out Auto-GEMS superficially, quickly and without thinking too much about the answers; 5% declared that someone explained some questions to them; only 1% declared that a person suggested some answers.

## Discussion

This manuscript describes the main features of Auto-GEMS, a newly developed, web-based, self-administered test for cognitive screening. Auto-GEMS includes 11 tasks that quickly provide a global estimate of an individual’s level of cognitive functioning in a short time (its completion requires about ten minutes). It collects examinees’ demographic information, such as age, education and sex, and it also measures their CR, which is a very reliable predictor of overall cognitive performance [29]. Auto-GEMS was adapted from two previously published cognitive screenings: GEMS [27], which is a paper-and-pencil test to be administered in person, and Tele-GEMS [28], which has to be administered at distance, via telephone or videoconference.

Auto-GEMS does not require any dedicated software as it can be accessed by any computer connected to the Internet via a web browser. Data collection for the majority of participants occurred only remotely. They received the link to perform the screening by personal email or the email of a relative/acquaintance. In such modality we collected the normative data of more than 1300 persons with different ages and educational background. In a sub-sample of participants we measured Auto-GEMS test-retest reliability, and checked its correlation with GEMS (paper-and-pencil) and Tele-GEMS (remote).

Overall, Auto-GEMS showed high internal consistency (good correlation among items and global score), satisfactory convergent validity (correlation with GEMS), high test-retest reliability, and equivalence with a parallel version (Auto-GEMS-B). The effect of demographic variables was investigated by using linear regressions. Respondents who were younger, more educated and with higher CR obtained the best scores. The modulation exerted by these variables was expected as it is a standard finding in cognitive testing. Such results are consistent with the literature reporting a strong relationship between life-experience CR proxies and global cognitive efficiency (e.g., Delgado-Losada et al., [14]. We, therefore, used age, sex, education and CR as predictors of the clinical cut-offs so as to guarantee a more accurate interpretation of test scores [10]. Following standard practice, performance is considered below the cut-off when the observed score is significantly lower than the predicted one. Along with the description of the test we provided the digital sources to use it in the different functioning modalities it allows along and a shiny app to compare the individual outcome with the normative data.

Auto-GEMS shows psychometric properties that suggest a good degree of accuracy in measuring a person’s global cognitive functioning. In addition, the method we used to calculate thresholds for significant changes takes into account the practice effect, allowing us to understand to what extent the second measure is significantly different from the first. In general, our participants showed a positive attitude in performing the test (data reported in the Feedback section), although the less expert ones had, when necessary, the help of someone either to check their email for obtaining the testing link or turn on the computer or explain to them how to use the mouse or keyboard.

Computer-based, self-administered online tests like Auto-GEMS can be seen as flexible tools which can provide useful information in a variety of contexts. While it is clear that we are only at the beginning of the development of self-administered screening tests, their clinical potential seems substantial. For instance, due to their characteristics they can be administered in a variety of contexts, also beyond those more commonly used for paper-and-pencil tests. Finally, as the self-administration of Auto-GEMS can be carried out in a familiar setting, examinees may feel less anxious as they are using their own device in their own home [27, 34]. This can help to measure more accurately their actual cognitive ability in their daily environment. Online digital tools might become particularly useful, for instance, to easily monitor over time the cognitive performance of the general population or of persons at risk of developing a specific neuropsychological disorder (e.g., for people with Subjective Cognitive Decline; Jessen et al., [17]. In the future, we might be able to use this approach to capture individual cognitive profiles and measure individual cognitive trajectories with particular reference to tasks which are sensitive to ageing, be it physiological or pathological. Of particular clinical interest would be the identification of relatively age-invariant cognitive measures to create an individual baseline for specific comparisons [8, 9]. Potential applications may include follow-up studies as well as large epidemiological screenings for the prevention and early detection of clinical decline. Moreover, the possibility of collecting reaction times might allow a more accurate measure for aspects of cognition which are often not routinely assessed.

Auto-GEMS can be administered both online or offline, either with or without data being sent to a remote server. This flexibility of use is further expanded by the availability of all its digital materials allowing to freely access and administered the task as well as performing a comparison with normative data, which are also made available in their integrity.

It is important to reiterate that Auto-GEMS can also be used in face-to-face clinical settings when it is informative to observe how the examinee interacts autonomously with the computer. This would follow the principles promoted by Singh and Germine [35] for a hybrid model of cognitive assessment, in which the integration of information gathered with different instruments and modalities improves the quality of evaluation and diagnosis.

As all computer-based tools, Auto-GEMS presents some limitations. Firstly, it requires a certain level of computer knowledge from participants, and it diminishes control over evaluation when completed without supervision.

In light of these considerations, it is important to emphasise that self-administered testing with Auto-GEMS cannot replace the need for in-person neuropsychological evaluation. Rather, it should be viewed as a valuable complementary tool, especially when considering the accessibility and convenience it offers for preliminary assessment and in longitudinal studies. The controlled and wide-ranging nature of in-person evaluations remains essential for the comprehensive understanding of a person’s cognitive status. However, the development of computer-based self-administered tests like Auto-GEMS is crucial to allow the implementation of the hybrid mode [35].

Notwithstanding the satisfactory outcome of this first data collection using Auto-GEMS, future investigations on its application in research and clinical settings are needed. As already mentioned above, in principle Auto-GEMS could effectively serve as a preliminary screening tool for clinical populations, particularly those with mild cognitive impairment (MCI) or early stages of dementia. Future research should therefore test participants with clinical conditions to confirm the applicability of Auto-GEMS to these populations. Future studies should also investigate the sensitivity of Auto-GEMS in detecting cognitive decline in these groups, possibly comparing performance with in-person assessments and/or with other remote testing tools.

Another potential direction could be the widening of the demographic diversity of the sample (e.g., different languages and cultural settings) in order to broaden its applicability.

Also, integrating Auto-GEMS with other digital health tools and platforms could enhance its utility by combining, for instance, cognitive screening data with lifestyle and health information thereby providing a more comprehensive assessment of an individual’s cognitive health. Such integration could facilitate personalised interventions and approaches to the monitoring and management of cognitive health.

## Electronic supplementary material

Below is the link to the electronic supplementary material.

Supplementary Material 1

## Data Availability

All data and materials are available on the OSF: https://osf.io/fq8g7/.

## Footnotes

1 TMT-A and TMT-B: The weighted scores of TMT-A and TMT-B are calculated by taking into account accuracy and processing speed. Their accuracy is weighted as the other tasks, while for processing speed we consider the empirical quantiles of the distribution of normative sample to assign a score from 0 to 9 (e.g., with a TMT-B score from 0 to 14.6 sec participants obtain 9, from 14.7 to 17.2 participants obtain 8, and so on until 54.8 sec and more, whereby participants obtain 0). To combine the weighted accuracy and processing speed we used this formula: (TMT_Accuracy*TMT_processing speed)/9.
