# Supplementary material for: Auto-Global Examination of Mental State (Auto-GEMS): a web-based self-administered cognitive screening
Source: Aging Clin Exp Res. 2024 Nov 11;36(1):216. doi: 10.1007/s40520-024-02862-z (PMC11554820; doi:10.1007/s40520-024-02862-z)
Supplement: Supplementary file 1 — Supplementary Material 1 [file 40520_2024_2862_MOESM1_ESM.docx]

**Auto-Global Examination of Mental State (Auto-GEMS): A web-based self-administered cognitive screening**

**Veronica Pucci*^1,2^, Giulio Contemori*^3^, Maria Silvia Saccani^3,4,5^, Giorgio Arcara^3,5^,**

**Sara Mondini^1,2,5^ and Mario Bonato*^3,4^**

**^1^**Department of Philosophy, Sociology, Education and Applied Psychology and Department of Developmental and Social Psychology and Socialization, University of Padua, Italy

**^2^**Human Inspired Technology Centre (HIT), University of Padua, Italy

**^3^**Department of General Psychology, University of Padua, Italy

**^4^**Padua Neuroscience Centre, University of Padua, Italy

**^5^**IRCSS, San-Camillo Hospital, Venice, Italy

Corresponding Authors:

Veronica Pucci: [veronica.pucci@phd.unipd.it](mailto:veronica.pucci@phd.unipd.it)

Giulio Contemori: [giulio.contemori@unipd.it](mailto:giulio.contemori@unipd.it)

Mario Bonato: [mario.bonato@unipd.it](mailto:mario.bonato@unipd.it)

**Supplementary Table 1 - Significant changes**

Supplementary Table 1 can be used to determine whether a significant change has occurred in the same individual after a second administration of Auto-GEMS-A. To do so it is necessary to identify the value observed in the first administration (central column) and check the corresponding row in the table. The values reported on the right side indicate the scores (for both Auto-GEMS-A and Auto-GEMS-B) above which a significant improvement may be inferred. The values reported on the left side of the central column indicate the scores below which a significant worsening may be assumed (for both Auto-GEMS-A and Auto-GEMS-B). A significant change is inferred if the score from the second measurement falls outside these thresholds. No significant change is inferred if the observed value falls within these thresholds, including threshold values. According to this approach, if no change has occurred in the second measurement, less than 5% of the population will exceed the upper thresholds, and less than 5% will fall below the lower thresholds. The grey cell values are predicted by the model and not observed in our sample. When the thresholds cannot be calculated, such as when the predicted value exceeds the range of possible score values, a hyphen is reported.

| Lower threshold with Auto-GEMS-B | Lower threshold with Auto-GEMS-A | **Observed value 1st administration**  **Auto-GEMS-A** | Upper threshold with Auto-GEMS-A | Upper threshold with Auto-GEMS-B |
| --- | --- | --- | --- | --- |
| - | - | **0** | 24.7 | 68.3 |
| - | - | **2** | 26.4 | 69.2 |
| - | - | **4** | 28.1 | 69.9 |
| - | 1.9 | **6** | 29.7 | 70.7 |
| 1.6 | 3.8 | **8** | 31.4 | 71.5 |
| 3.1 | 5.7 | **10** | 33 | 72.4 |
| 4.5 | 7.7 | **12** | 34.7 | 73.2 |
| 5.9 | 9.6 | **14** | 36.4 | 74 |
| 7.3 | 11.5 | **16** | 38 | 74.8 |
| 8.7 | 13.4 | **18** | 39.7 | 75.7 |
| 10.1 | 15.3 | **20** | 41.4 | 76.5 |
| 11.4 | 17.2 | **22** | 43.1 | 77.4 |
| 12.8 | 19.1 | **24** | 44.8 | 78.2 |
| 14.2 | 21 | **26** | 46.4 | 79.1 |
| 15.5 | 22.9 | **28** | 48.1 | 79.9 |
| 16.9 | 24.7 | **30** | 49.8 | 80.8 |
| 18.2 | 26.6 | **32** | 51.5 | 81.7 |
| 19.5 | 28.5 | **34** | 53.2 | 82.6 |
| 20.9 | 30.4 | **36** | 54.9 | 83.6 |
| 22.2 | 32.3 | **38** | 56.6 | 84.5 |
| 23.5 | 34.1 | **40** | 58.3 | 85.4 |
| 24.7 | 36 | **42** | 60 | 86.4 |
| 26 | 37.9 | **44** | 61.8 | 87.3 |
| 27.3 | 39.7 | **46** | 63.5 | 88.3 |
| 28.6 | 41.6 | **48** | 65.2 | 89.2 |
| 29.8 | 43.4 | **50** | 66.9 | 90.2 |
| 31 | 45.3 | **52** | 68.7 | 91.2 |
| 32.3 | 47.1 | **54** | 70.4 | 92.2 |
| 33.5 | 49 | **56** | 72.1 | 93.2 |
| 34.7 | 50.8 | **58** | 73.9 | 94.3 |
| 35.9 | 52.6 | **60** | 75.6 | 95.3 |
| 37.1 | 54.4 | **62** | 77.4 | 96.3 |
| 38.2 | 56.3 | **64** | 79.1 | 97.4 |
| 39.4 | 58.1 | **66** | 80.9 | 98.5 |
| 40.6 | 59.9 | **68** | 82.7 | 99.6 |
| 41.7 | 61.7 | **70** | 84.4 | 100 |
| 42.8 | 63.5 | **72** | 86.2 | - |
| 43.9 | 65.3 | **74** | 88 | - |
| 45.1 | 67.1 | **76** | 89.8 | - |
| 46.1 | 68.9 | **78** | 91.5 | - |
| 47.2 | 70.7 | **80** | 93.3 | - |
| 48.3 | 72.5 | **82** | 95.1 | - |
| 49.4 | 74.3 | **84** | 96.9 | - |
| 50.4 | 76.1 | **86** | 98.7 | - |
| 51.4 | 77.8 | **88** | 100 | - |
| 52.5 | 79.6 | **90** | - | - |
| 53.5 | 81.4 | **92** | - | - |
| 54.5 | 83.1 | **94** | - | - |
| 55.5 | 84.9 | **96** | - | - |
| 56.5 | 86.6 | **98** | - | - |
| 57.5 | 88.4 | **100** | - | - |

**Supplementary Table 2 - Feedback from participants**

| 1 | *I have carried out the task to the best of my ability.* |
| --- | --- |
| 2 | *I have been interrupted or distracted during the task.* |
| 3 | *I carried out the task quickly, without focusing on my answers.* |
| 4 | *I have carried out the task autonomously from start to finish.* |
| 5 | *A person opened the email for me, and then I carried out the task autonomously.* |
| 6 | *I carried out the task by answering the questions autonomously, but a person helped me to use the mouse and the keyboard.* |
| 7 | *I carried out the task with a person who explained some questions to me.* |
| 8 | *I carried out the task with a person who suggested some of the answers to me.* |
